# The value of ultrasonography combined with carbohydrate antigen 125 and 19-9 detection in the diagnosis of borderline ovarian tumors and prediction of recurrence

**DOI:** 10.3389/fsurg.2022.951472

**Published:** 2023-01-06

**Authors:** Lina Niu, Weibin Wang, Yongjun Xu, Tao Xu, Jiali Sun, Weiqin Lv, Junli Zhang, Lirong Qiu, XuFeng Dong, Yun Shang, Lizhen Zhang, Junxia Wang

**Affiliations:** ^1^ Department of Gynecology, Yuncheng Central Hospital, Yuncheng, China; ^2^ Department of Pharmacy, Yuncheng Central Hospital, Yuncheng, China; ^3^ Department of Pathology, Yuncheng Central Hospital, Yuncheng, China; ^4^ Department of Ultrasound, Yuncheng Central Hospital, Yuncheng, China; ^5^ Department of Infectious Disease Prevention and Control Division, Shanxi Center for Disease Control and Prevention, Taiyuan, China

**Keywords:** CA 125, CA 19-9, borderline ovarian tumors, recurrence, differential diagnosis, ultrasonography

## Abstract

**Objective:**

This study aimed to investigate the clinical value of ultrasonography combined with tumor markers in the diagnosis and prediction of recurrence of borderline ovarian tumors (BOTs) and analyze the value of the combination of two different auxiliary examinations in the diagnosis and prediction of recurrence of BOTs.

**Methods:**

Here, 221 patients with BOTs confirmed by postoperative pathology were enrolled. Their clinical data, including the ultrasonography features, tumor markers, and clinicopathological data, were retrospectively analyzed.

**Results:**

The statistical data of the 221 cases with BOTs were as follows: 94 (42.5%) with left-sided lesions, 102 (46.2%) with right-sided lesions, and 25 (11.3%) with bilateral lesions. Moreover, 93 cases (42.1%) had a borderline serous tumor, 110 (49.8%) had a borderline mucinous tumor, 12 (5.4%) had a borderline serous mucinous tumor, 2 (0.9%) had a borderline endometrioid tumor, 1 (0.5%) had a borderline Brenner tumor, and 2 (0.9%) had a clear cell BOT. There were 104 cases (47.1%) with a tumor diameter of ≤10 cm and 117 cases (52.9%) with a tumor diameter of >10 cm as suggested by ultrasonography. There were 89 cases (40.3%) with septation, 44 (19.9%) with papilla, and 97 (43.9%) with blood flow as demonstrated by ultrasonography. Carbohydrate antigen 125 (CA 125) was elevated in 132 cases (59.7%), and CA 19-9 was elevated in 52 cases (23.5%).

**Conclusion:**

In general, BOTs are difficult to diagnose preoperatively and have a certain recurrence rate. Ultrasonography combined with CA 125 and CA 19-9 is significant for the preoperative diagnosis and selection of surgical modality for BOTs and could be used as a guideline to achieve good preoperative preparation and avoid secondary surgery.

## Introduction

The pathological types of borderline ovarian tumors (BOTs) vary greatly (1). They have certain recurrence and mortality rates, and there are difficulties in differentiating BOTs from benign ovarian and malignant tumors, difficulties in the preoperative and intraoperative diagnosis, and the risk of excessive trauma, secondary surgery, and postoperative recurrence in patients. The ultrasound is certainly a cost-effective exam and the role of ultrasound is well defined in primary diagnosis of ovarian tumors. Although it is still controversial during follow-up of surgically treated ovarian tumors, the ultrasound is an efficient tool in the early detection of ovarian tumors (2, 3).

In the present study, the general clinical data of 221 patients were retrospectively analyzed, and the ultrasonography characteristics of the BOTs, the CA 125 and CA 19-9 values for the preoperative diagnosis of the disease, and the prediction of the recurrence were analyzed in depth in combination with a literature analysis (4, 5). The value of the combined application of ultrasonography and CA 125/CA 19-9 detection in the diagnosis and prediction of the disease's recurrence was also discussed.

## Materials and methods

### Materials

A total of 221 patients with ovarian tumors admitted to our hospital from January 2010 to December 2018 were selected for the retrospective analysis. All patients were diagnosed with BOTs by postoperative pathological examination with complete ultrasonography and clinicopathological data. They had a minimum age of 13 years and a maximum age of 84 years, with a mean age of 43.20 ± 14.86 years. All patients were treated surgically, and relevant pathological histological findings were obtained from the postoperative specimens. This study was approved by the Ethics Committee of Yuncheng Central Hospital (No. YXLL 2022011-1).

### Methods

Transabdominal ultrasonography, transvaginal ultrasonography, a combination of transabdominal and transvaginal ultrasonography, and other gynecologic ultrasonography examinations were conducted in all patients. The carbohydrate antigen 125 (CA 125) and CA 19-9 detections were conducted in the hospital. The patients’ sonographic characteristics were analyzed, including the number of masses (unilateral and bilateral), location (left-sided, right-sided, and pelvis), size, morphology (especially the internal translucency of the masses), and the presence or absence of septation and papillae. The tumor markers, postoperative pathological examination results, and recurrence were also examined.

## Results

Among the 221 enrolled cases, there were 94 (42.5%) with left-sided lesions, 102 (46.2%) with right-sided lesions, and 25 (11.3%) with bilateral lesions. The detailed pathological results and tumor type distribution are demonstrated in Table 1. Compared with other pathological types, borderline serous tumors were more likely to be bilaterally involved (i.e., 60.0% of the serous tumors, 36.0% of the mucinous tumors, and 4.0% of the serous mucinous tumors). There was no statistically significant difference in the tumor type distribution (*P* > 0.05). There were 89 cases (40.3%) with septation, 44 cases (19.9%) with papilla, and 97 cases (43.9%) with blood flow as demonstrated by ultrasonography.

**Table 1:** The pathological data of 221 patients with borderline ovarian tumors (cases and percentages).

Tumor location	Serous	Mucinous	Serous mucinous	ovarian endometrioid	Brenner	Clear cell tumor	Migrating cells	Total
Left-sided	32 (34.0)	58 (61.7)	2 (2.1)	1 (1.1)	0	1 (1.1)	0	94
Right-sided	46 (45.1)	43 (42.2)	9 (8.8)	1 (1.0)	1 (1.0)	1 (1.0)	1 (1.0)	102
Bilateral	15 (60.0)	9 (36)	1 (4.0)	0	0	0	0	25
Total	93 (42.1)	110 (49.8)	12 (5.4)	2 (0.9)	1 (0.5)	2 (0.9)	1 (0.5)	221

The ultrasonography features comprised cystic solid masses (i.e., regular morphology), clear cystic walls, and cystic textures. Several BOTs had visible septation, most with visible blood flow, and mainly manifested as multifocal masses. The ultrasound sonogram results, tumor markers, and pathological features are shown in Table 2.

**Table 2:** The ultrasound sonogram features in 221 patients with borderline ovarian tumors.

		Serous	Mucinous	Serous mucinous	ovarian endometrioid	Brenner	Clear cell tumor	Migrating cells
The maximum diameter								
	Median (cm)	7.0	14.0	9.5	9.3	2.0	7.0	9.5
	≤10	69 (66.3)	26 (25.0)	6 (5.8)	1 (1.0)	1 (1.0)	1 (1.0)	0
	>10	24 (20.5)	84 (71.8)	6 (5.1)	1 (0.9)	0	1 (0.9)	1 (0.9)
Papillae								
	With	28 (63.6)	15 (34.1)	1 (2.3)	0	0	0	0
	Without	65 (36.7)	95 (53.7)	11 (6.2)	2 (1.1)	1 (0.6)	2 (1.1)	1 (0.6)
Separation								
	With	14 (15.7)	69 (77.5)	5 (5.6)	1 (1.1)	0	0	0
	Without	79 (59.8)	41 (31.1)	7 (5.3)	1 (0.8)	1 (0.8)	2 (1.5)	1 (0.8)
Texture								
	Cystic	38 (314)	72 (59.5)	8 (6.6)	1 (0.8)	1 (0.8)	1 (0.8)	0
	Solid	4 (80.0)	1 (20.0)	0	0	0	0	0
	Systic and solid	51 (53.7)	37 (38.9)	4 (4.2)	1 (1.1)	0	1 (1.1)	1 (1.1)
Blood flow signal								
	With	36 (37.1)	54 (55.7)	4 (4.1)	1 (1.0)	0	1 (1.0)	1 (1.0)
	Without	57 (46.0	56 (45.2)	8 (6.5)	1 (0.8)	1 (0.8)	1 (0.8)	0

Among the 221 patients, 132 (59.7%) had elevated CA 125 levels, and 89 (40.3%) had normal CA 125 levels; the CA 19-9 level was elevated in 52 cases (23.5%), while 169 (76.5%) had normal CA 19-9 levels. The details are illustrated in Table 3.

**Table 3:** The changes of CA125 and CA199 in patients with borderline ovarian tumors.

		Serous	Mucinous	Serous mucinous	ovarian endometrioid	Brenner	Clear cell tumor	Migrating cells
CA125								
	Normal	35 (39.3)	50 (56.2)	3 (3.4)	1 (1.1)	0	0	0
	Elevation	58 (43.9)	60 (45.5)	9 (6.8)	1 (0.8)	1 (0.8)	2 (1.5)	1 (0.8)
CA199								
	Normal	79 (46.7)	76 (45.0)	8 (4.7)	2 (1.2)	1 (0.6)	2 (1.2)	1 (0.6)
	Elevation	14 (26.9)	34 (65.4)	4 (7.7)	0	0	0	0

## Discussion

In 1973, the World Health Organization (WHO) officially termed ovarian tumors as borderline malignant tumors (i.e., carcinoma with low malignant potential) or low-grade potentially malignant tumors in the histological classification of ovarian tumors (6). The term borderline/atypical proliferative tumor was adopted to designate this tumor type, which was previously referred to as low malignant potential tumor (7) in the 2014 edition of *WHO Classification of Tumors of the Female Genital Organs* (8). Currently, it is difficult to confirm the preoperative diagnosis of BOTs, and the confirmation of a specific diagnosis needs to be combined with clinical manifestations, imaging examinations, tumor marker detection, etc. An intraoperative frozen section can assist in the diagnosis, but the final confirmation still relies on pathological examination.

In many studies, BOTs are assigned to a special manifestation of malignant tumors. According to epidemiological research, the clinical incidence of BOTs is increasing yearly, as is the proportion of malignant tumors, which is directly related to improvements in pathology and ultrasonography diagnosis technology and the application of tumor markers to a certain extent (9). In the present study, approximately 62.9% of patients had no obvious symptoms of discomfort and requested surgery due to pelvic masses found by physical examination.

Here, 93 cases (42.1%) had a borderline serous tumor, 110 (49.8%) had a borderline mucinous tumor, 12 (5.4%) had a borderline serous mucinous tumor, 2 (0.9%) had a borderline endometrioid tumor, 1 (0.5%) had a borderline Brenner tumor, and 2 (0.9%) had a clear cell BOT. The ultrasonography results demonstrated that 104 cases (47.1%) had a tumor diameter of ≤10 cm and that 117 (52.9%) had a tumor diameter of >10 cm. There were 89 cases (40.3%) with septation, 44 (19.9%) with papilla, and 97 (43.9%) with blood flow as demonstrated by ultrasonography. In the laboratory examination, CA 125 was elevated in 132 cases (59.7%), and 89 cases (40.3%) had normal CA 125 levels. In general, BOTs have greatly varied biological behavior and diverse clinical manifestations that are difficult to accurately diagnose preoperatively, and their prognosis is significantly correlated with the surgical approach taken. A previous study suggested that 65% of patients are <40 years old (10), that the tumors are increasing in the young population, and that the patients are mainly women of reproductive age. Most patients with BOTs may achieve a good prognostic outcome with a high five-year survival rate, and the risk of recurrence and metastasis exists in only a few cases (4). In clinical treatment, it is important to reduce the recurrence rate and maximize the preservation of patients’ reproductive function, and unnecessary surgical trauma should be avoided, which is the key to the diagnosis and treatment of BOTs. Impairing autophagic flux could be a novel strategy of cancer therapy (11). However, controversies still exist.

The preoperative identification of pelvic masses mainly relies on gynecologic ultrasonography and the detection of tumor markers, and intraoperative diagnosis mainly relies on intraoperative frozen section examination. Previous study has shown that the ovarian cancer has some specific ultrasound characteristics (12). However, BOTs and ovarian tumors have similar ultrasonographic features, it is difficult to accurately distinguish BOTs from other ovarian tumors (13). Papillary protrusion is a characteristic manifestation of BOTs. In ovarian tumors, whether benign or malignant, the possibility of papilla is high, and as the risk of malignancy increases, the possibility of irregular papilla and papilla with an increased volume increases accordingly. In several cases with borderline serous tumors, septation occurs, and small papillae may appear on the septation. In contrast, borderline mucinous tumors are more likely to have septal-like structures, and the tumor size is significantly larger (13). Once a typical papillary ultrasound presentation is found, the possibility that the patient has a borderline tumor cannot be ruled out. A serous cystic adenoma is more likely to have a similar ultrasound presentation, making it difficult to accurately diagnose BOTs and leading to false positivity. However, for women of childbearing age, considering the reproductive function and avoiding secondary surgery, there is a need for active early diagnosis and treatment. Moreover, the risks of preoperative and intraoperative misdiagnosis and missed diagnosis should be minimized. Additionally, the surgical staging is mainly pursued *via* a minimally invasive approach in case of BOTs or apparently early stage ovarian cancer. Minimally invasive approach is associated with reduced hospitalization, fewer post-operative complications and better cosmetic results (14, 15).

In a previous study concerning the ultrasound presentation and CA 125 level in BOTs and invasive tumors, univariate and multivariate logistic regression analyses were used to investigate the independent risk factors for BOTs, mainly including the tumor size, solid tumor component, septation, and CA 125 level (16). It was found that the specificity of CA 125 was not high but was able to sensitively determine the extent of disease involvement. Based on this, a scientific assessment of disease progression and an accurate prediction of tumor recurrence could be achieved. Ultrasonography diagnosis of an ovarian tumor requires continuous and dynamic observation of the tumor as a whole. It has been shown that BOTs’ histological type, size, and location (unilateral or bilateral) correlate with the accuracy of intraoperative frozen sections (17). The present study also had to provide the pathologist with a detailed medical history, ultrasonography findings, tumor markers, and intraoperative findings when sending the frozen sections. Upon retrospectively analyzing the ultrasonography images of the 221 patients included here, most sonographers made descriptive diagnoses, such as “cystic mass” and “multifocal cystic mass,” except for a few, who suggested the diagnosis of BOTs. Therefore, it could be inferred that the diagnosis of BOTs is difficult for most ultrasonographers. Transabdominal ultrasound often has difficulty in timely detecting micro-papillary structures in the wall, whereas transvaginal ultrasound can effectively identify and diagnose BOTs. The results of the multivariate analysis revealed that the tumor size and cystic solidity and the CA 125 level are clinically significant in predicting tumor recurrence, suggesting that ovarian tumors with a diameter >10 cm, cystic solidity, or solidity on ultrasonography and elevated CA 125 levels have a high risk of postoperative recurrence. The details are demonstrated in Tables 4, 5. For patients with these characteristics, it is recommended to undergo comprehensive staged surgery and enhanced follow-up. The CA 125 level can be adopted as a screening and postoperative review monitoring index for patients with BOTs, and great importance should be attached to patients with elevated CA 125 levels (18, 19).

**Table 4:** The chi-square analysis of the ultrasonography characteristics, levels of CA125 and CA199 and recurrence in patients.

Factor	*N* (%)	Recurrence		*χ* ^2^	*P*
		With	Without		
B-ultrasonography (the maximum tumor diameter)	221			4.846	0.028
≤10 cm	104 (47.1)	4	100		
>10 cm	117 (52.9)	14	103		
The tumor involvement in the ovarium	221			0.691	0.790
Left-sided	94 (42.5)	8	86		
Right-sided	102 (46.2)	7	95		
Bilateral	25 (11.3)	3	22		
B-ultrasonography (with or without separation)	221			5.676	0.017
With	89 (40.3)	12	77		
Without	132 (59.7)	6	126		
B-ultrasonography (with or without papillae)	221				0.538*
With	44 (19.9)	2	42		
Without	177 (80.1)	16	161		
The blood flow in B-ultrasonography	221			4.127	0.042
With	97 (43.9)	12	85		
Without	124 (56.1)	6	118		
The texture in B-ultrasonography	221		0.079		0.969*
Normal	121 (54.8)	10	111		
Elevation	5 (2.3)	0	5		
Normal	95 (42.9)	8	87		
CA125	221			4.698	0.03
Normal	89 (40.3)	3	86		
Elevation	132 (59.7)	15	117		
CA199	221				0.772*
Normal	169 (76.5)	13	156		
Elevation	52 (23.5)	5	47		

* The accurate probability method.

**Table 5:** The logistic regression analysis of the influencing factors for the recurrence of borderline ovarian tumors.

	*B*	Wals *χ*^2^	*P*	Exp. (*B*)	95% confidential interval
The level of CA125 (normal or abnormal)	1.330	4.141	0.042	3.783	1.050–13.624
Ultrasonography (with or without separation)	1.201	5.209	0.022	3.324	1.185–9.325
The constant	1.461	18.497	0	4.312	

In the present study, the clinical characteristics, tumor markers, ultrasonography features, and recurrence rates of BOTs were analyzed, but the recurrence of different pathological tumor types was not studied in detail. This was a retrospective study with some missing data, a small sample size, and a short follow-up duration for some of the patients, so long-term follow-up is needed for good clinical analysis in future studies. In future work, in-depth, careful analysis and discussion of the ultrasonography features, levels of CA 125 and CA 19-9, and prognostic features of BOTs with different pathological subtypes should also be conducted.

In summary, although it is difficult to accurately diagnose BOTs preoperatively, ultrasonography can provide an accurate and detailed description of the characteristics of the masses, and combined with the levels of tumor markers, it can indicate benignity or malignancy. Therefore, it is easier and more accurate to judge by the intraoperative frozen sections according to the preoperative indications. Patients’ general condition and ultrasonography features are also valuable in predicting the recurrence rate and can aid in the surgical approach selection, postoperative monitoring, and follow-up (20), minimizing the trauma caused by secondary surgery and reducing unnecessary recurrence and death caused by recurrence.

## Data Availability

The original contributions presented in the study are included in the article/Supplementary Material, further inquiries can be directed to the corresponding author/s.
